# Advances in AI for web integrity, equity, and well-being

**DOI:** 10.3389/fdata.2023.1125083

**Published:** 2023-05-05

**Authors:** Srijan Kumar

**Affiliations:** Georgia Institute of Technology, Atlanta, GA, United States

**Keywords:** artificial intelligence, applied machine learning, data mining, social networks, misinformation, bad actors

## Abstract

My research develops data mining, AI, and applied machine learning methods to combat malicious actors (sockpuppets, ban evaders, etc.) and dangerous content (misinformation, hate, etc.) on web platforms. My vision is to create a trustworthy online ecosystem for everyone and the next generation of socially-aware methods that promote health, equity, and integrity of users, communities, and platforms online. Broadly, in my research, I create novel graph, content (NLP, multimodality), and adversarial machine learning methods leveraging terabytes of data to detect, predict, and mitigate online threats. My interdisciplinary research innovates socio-technical solutions that I achieve by amalgamating computer science with social science theories. My research seeks to start a paradigm shift from the current slow and reactive approach against online harms to agile, proactive, and whole-of-society solutions. In this article, I shall describe my research efforts along four thrusts to achieve my goals: (1) Detection of harmful content and malicious actors across platforms, languages, and modalities; (2) Robust detection models against adversarial actors by predicting future malicious activities; (3) Attribution of the impact of harmful content in online and real world; and (4) Mitigation techniques to counter misinformation by professionals and non-expert crowds. Together, these thrusts give a set of holistic solutions to combat cyberharms. I am also passionate about putting my research into practice—my lab's models have been deployed on Flipkart, influenced Twitter's Birdwatch, and now being deployed on Wikipedia.

## 1. Introduction

I develop data mining methods to detect, predict, and mitigate the threats to integrity, equity, and well-being posed by online malicious actors and harmful content. Bad actors (e.g., evaders, sockpuppets, fraudsters, fake reviewers, vandals, trolls, etc.) and content (e.g., misinformation, hate speech, fake reviews, etc.) pose one of the biggest threats to today's public health, democracy, science, and society. Actions in the cyber sphere have severe consequences on real-life decisions. For example, online hate speech against racial minorities leads to exacerbating the mental health of victims and leads to physical violence. Moreover, mis/dis/mal-information reduces the trust in vaccines and health policies leading to preventable deaths and incites violence and harassment against racial minorities. Furthermore, state-sponsored entities use sockpuppet accounts to promote propaganda and increase polarization leading to a fractured society. Online harms disproportionately impact marginalized communities both online and in the real world, which lead to inequities across the society. Together, online harmful content and bad actors harm platform integrity, user well-being, and community equity.

My research vision is to enable a future where online users can have healthy social interactions, online platforms provide trustworthy information, AI models are secure and reliable, and malicious actors cannot manipulate AI models for their nefarious goals. To combat harmful online threats, I develop efficient, robust, and scalable models to detect, predict, and mitigate online threats. I innovate social networks, graph neural networks, natural language processing techniques, and leverage adversarial learning methods while utilizing terabytes of data. My research is interdisciplinary. I leverage social science theories and amalgamate it with large-scale computation from computer science to develop socio-technical solutions. Notably, I seek to change the current paradigm of web integrity from being slow and reactive to bad actors to being agile and proactive.

Currently, I direct the CLAWS Data Science research group at the College of Computing at Georgia Institute of Technology. Prior to joining Georgia Institute of Technology as an Assistant Professor in January, 2020, I worked on creating accurate, efficient, and scalable methods for web integrity and safety. My research interest in this domain started during my Ph.D. at the University of Maryland, accelerated during my postdoctoral training at Stanford University, and was scaled up during my role as a Visiting Researcher at Google Research.

During these periods, my research focused on developing novel models spanning graphs (Kumar et al., 2014, 2016a, 2018b; Kumar, 2016), text (Kumar et al., 2015, 2016b; Evans, 2018), and behavior modeling (Kumar et al., 2017, 2018a). My work focused on the following application tasks: detecting sockpuppets on multiple online discussion platforms (Kumar et al., 2017), vandals on Wikipedia (Kumar et al., 2015), trolls on Slashdot (Kumar et al., 2014), inter-community harassment (Kumar et al., 2018a), hoaxes on Wikipedia (Kumar et al., 2016b), and fake reviews on e-commerce platforms (Kumar et al., 2018b).

The COVID-19 pandemic altered the focus of my research shortly. In March and April 2020, I witnessed first-hand and in real-time the mass panic, confusion, violence, and harmful impact of dangerous content and malicious actors online. I saw that online social media platforms were rampant with misinformation regarding COVID-19 - false claims of various fake cures (eating garlic, drinking lemon water, etc.), blaming 5G and Bill Gates for causing COVID-19—and the challenge the society faced in distinguishing truth from falsehoods. Simultaneously, hateful rhetoric against Asians were omnipresent along with incidents of physical assault and violence against Asians. The nefarious activities of malicious actors were being amplified by AI algorithms and recommender systems were serving conspiracy theories to people.

These events propelled me to leverage my research experience and focus my research on developing data-driven trustworthy AI models that can accurately and robustly combat online bad actors and harmful content. These resulted in development of new methods spanning robust detection and prediction models (He et al., 2021a; Mujumdar and Kumar, 2021; Oh et al., 2022b; Verma et al., 2022c), advance natural language processing and multimodality (Verma et al., 2022b,c), graphs (Raghavendra et al., 2022; Sharma et al., 2022), and recommender systems (Oh et al., 2021, 2022a; Shalaby et al., 2022). The applications of these methods were in web integrity and led to new findings about online health misinformation (Micallef et al., 2020, 2022; Verma et al., 2022a), counter-misinformation (Micallef et al., 2020; He et al., 2023; Ma et al., 2023), hate speech (He et al., 2021b), ban evaders (Niverthi et al., 2022), and digital deception (Glenski et al., 2020; Kumar et al., 2021).

My research is multi-disciplinary where I work closely with academic collaborators in social science, communication science, journalism, and public health. I seek to help domain experts and various stakeholders solve on-the-ground problems by developing efficient AI, machine learning, and data mining techniques.

I am deeply passionate about bringing my research into practice. Therefore, I partner with end users, including professional fact-checkers (PolitiFact, AFP) and online moderators on Wikipedia, as well as a non-profit organization (Anti-Defamation League) for deep community engagement. First, a model I created to identify fake reviewers (Kumar et al., 2018b) was deployed at Flipkart (India's largest e-commerce platform) and showed improvement in early detection of fake reviews and reviews. Second, a recent research paper (Micallef et al., 2020) was an early influencer of Twitter's Birdwatch (a community-driven misinformation flagging system) by showing that misinformation is actively being countered by non-expert crowd users. Third, a model to identify ban evaders based on our recent paper (Niverthi et al., 2022) is currently being deployed to aid Wikipedia moderators identify ban evading malicious actors on the platform accurately and faster. Through multiple NSF-funded grants, I am building tools to empower non-expert users (He et al., 2023; Ma et al., 2023) and professionals to correct and counter online misinformation.

Platform integrity is incredibly **challenging** due to several reasons. First, online harms know no platform boundaries, spread in multiple languages, and use combinations of text, images, and videos to convey their harms. However, existing methods focus on single language, single platform, and single modality of content. Creating new methods that can extract meaningful signals from across these aspects is non-trivial. Second, bad actors use multiple accounts to hide their malicious activities. They evolve their strategies to evade detection by detectors. Existing methods are unable to account for such complex strategies of adversarial behavior and how models can remain effective in the face of these strategies. Third, it is non-trivial to estimate the impact that online harms have online or in the real world. Conducting surveys is expensive, often done at a small scale, and is time consuming; on the other hand, using large-scale online social media data is observational and mostly reflecting online behavior (opposed to real-world impact). Finally, for mitigation, a general set of generalizable mitigation strategies that work across user groups, platforms, and topics do not exist. Developing and deploying mitigation tools requires expertise to understand the pain points and needs of the end users (platform moderators, fact checkers, journalists), the network to connect with the end users, and have the capabilities to iteratively develop the tool to be deployed in practice. At the same time, technically, it is important to make the tool easy to user and provide the functionalities for machine learning models to explain their decisions to the non-technical end users.

Below I shall describe the four key research thrusts with which I seek to achieve comprehensive all-rounded solutions for web safety and integrity:

(1) **Detection**: “Multi-X” detection of harmful content and malicious actors across platforms, languages, and modalities;
(2) **Robustness**: Robust detection models against adversarial actors by predicting future malicious activities;
(3) **Attribution**: Attributing the impact of harmful content in online and real world and the role that recommender systems play;
(4) **Mitigation**: Developing mitigation techniques and tools to counter misinformation.

These four thrusts seek to develop complementary approaches to solve online integrity problems. Together, they create end-to-end framework to provide holistic solutions from detection all the way to mitigation. The first two thrusts ensure that the detection models are accurate as well as robust. The third thrust aims to estimate the impact that online harms have. And finally, the fourth thrust develops a series of solutions to counter online harms and mitigate its negative impact.

The solutions proposed in the four thrusts have the potential to improve integrity, equity, and well-being of users, communities, and platforms. With the methods and tools, bad actors and dangerous content can be detected, removed, and mitigated as early as possible, thus improving safety and integrity. Since marginalized communities are disproportionately harmed due to dangerous content and users, mitigating them helps improve equity. Finally, the well-being of users and communities degrades due to the negative influence of interactions with dangerous content and harmful users. Thus, mitigating their harms can improve the overall well-being.

Below I will describe the efforts in each of these thrusts.

## 2. “Multi-X” detection of harmful content and malicious actors across platforms, languages, and modalities

Most research is conducted on “English text on Twitter” due to the ease of data access and data collection. My research seeks to address the grand challenge of addressing fundamental problems rooted deeply across platforms, languages, and modalities including images, videos, and text. “Multi-X” stands for multi-platform, multi-lingual, and multi-modal models. With my students, I plan to achieve this by building better content-agnostic graph-based models and powerful Multi-X models.

### 2.1. Dynamic graph models

Using non-textual data, namely graph-based and user behavior modeling-based attributes, is a powerful solution to create language-agnostic and platform-agnostic models. Therefore, I have created novel dynamic graph-based methods that can learn representations from dynamically evolving networks. I created a scalable dynamic graph neural network model called JODIE (Kumar et al., 2019).

User actions can be modeled as a temporal interaction network between users and items, e.g., users interacting with posts on social media, where interactions can be writing, liking, commenting, etc.; in e-commerce, users interacting (purchasing, viewing, adding to cart, writing review) with products. The temporal interaction network represents timestamped interactions between users and items as the network having user nodes, item nodes, and interaction edges. Timestamps are associated in the edges along with interaction features.

Given a temporal interaction network, the model generates dynamically-evolving embedding trajectories of users and items. In contrast with the most frequently-used static embeddings, the advantage of dynamic embeddings is to model the temporal evolution of each user nodes and item nodes.

The model has two major components: update and project. The update component updates the user and item embeddings *after* interaction, while the project component predicts future embedding trajectory *before* interaction. Notably, this model was the first to predict future evolution of trajectories.

My collaborators and I used the JODIE model to detect malicious users on Wikipedia and Reddit from interaction networks between users and items (i.e., pages on Wikipedia and subreddits on Reddit). The improved performance arises from the distinct behavioral and interaction signatures between benign and malicious actors, both in terms of the who they interact with, when, and how frequently. JODIE outperformed several baselines spanning dynamic graph models and sequential models by a significant margin of 12% in this detection task. The code and data for this work is present at https://github.com/claws-lab/jodie.

My students and I have further extended the model to signed and dynamic network, where edges have a positive or negative relationship associated with it. The model, named SEMBA (Raghavendra et al., 2022), learns to incorporate the social science theory, called Balance Theory, which governs how signed edges are formed in the networks—specifically, edges are created to close triads in a balanced manner such that each triad has an even number of negative signed edge. This special semantics requires the model to learn distinct representations of positive vs. negative signs. To model this, SEMBA extends the TGN framework (Rossi et al., 2020) to learn positive and negative memories of nodes, which are updated following the rules of Balance Theory. The model is trained to effectively predict future signed relationships between nodes in the network.

### 2.2. Multi-platform models

Online harmful content spreads across platforms. My research has recently shown how misinformation spreading on Twitter, Reddit, and Facebook use YouTube videos as “evidence” to support the misinformation claims (Micallef et al., 2020, 2022).

In this work, my collaborators and I created a novel taxonomy regarding the use of YouTube videos (as an instance of “external evidence”) on the aforementioned social media platforms to spread online harms. Most notably, the taxonomy covered the different relationships between the content of posts on the social media platforms and the YouTube video—whether the video supports the post, the video is related to but does not support the post, the video contradicts the post, the video supports but is taken out-of-content, and finally, the video is unrelated to the post content. Other dimensions of posts and videos are also considered in the taxonomy—regarding the misinformation classification of post content, video content, and post-video content as a single unit.

A total of 3,000 social media posts and 991 YouTube videos were annotated as per the taxonomy. Following the annotations, BERT classifiers were trained which achieved an F-1 score and precision of over 0.74. The fine-grained taxonomy revealed new insights. Importantly, a major finding revealed that when YouTube videos are linked from misinformation posts, they are overwhelmingly used in support of the video (over 80% cases) while it is taken out of content between 3 and 11% times.

Notably, the detection performance of cross-platform misinformation improves significantly when using features from multiple platforms. The research showed how misinformation spreads across platforms and highlighted the need to leverage cross-platform data for enhanced predictive performance.

The cross-platform nature of the problem makes it challenging for individual platforms to combat online harms, since they only have a limited view of the harmful content within their own platform. This highlights the need to create cross-platform data sharing to enable platforms to counteract online harms early. Such multi-platform data sharing partnerships have been successfully established and operationalized to address high-stakes challenges such as counter-terrorism *via* the GIFCT partnership and combating CSAM (Child Sexual Abuse Material). I advocate the urgent need to create such partnership to address the challenge of online misinformation, hate speech, and other forms of online harms.

### 2.3. Multilingual models

About 75% of internet content is not in English and harmful content in low-resourced languages harms disadvantaged communities. My research seeks to go beyond the standard studies of creating English language model, but instead evaluate and improve models in non-English languages.

My students and I have recently shown that the current state-of-the-art natural language processing methods perform poorly in detecting harmful content in non-English languages, compared to detecting equivalent content in English (Verma et al., 2022b).

The key hypothesis this work is based on is that since the development of computational linguistic models has primarily focused on English, it is likely that models in other languages fall short on the same task but in a less-researched or lower-resourced language. To validate this hypothesis, popular models were evaluated on three tasks, namely crisis information, fake news, and emotion recognition.

For fair evaluation, equivalent content datasets were created in multiple languages by using manual as well as automated translations (which were then validated for quality). The studied languages include English, Spanish, Portuguese, French, Hindi, and Chinese.

**Figure 1:**
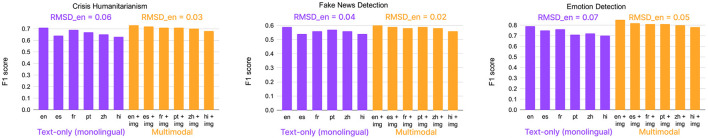
Comparing *F*_1_ scores on non-English and English text for both text-only and multimodal classifiers using *monolingual* language models. RMSD_*en*_ denotes the root-mean-square deviation of the *F*_1_ scores achieved by non-English classifiers with respect to that of the corresponding English classifier. The RMSD_*en*_ values for multimodal models are lower than those for monolingual text-only models.

Experiments showed that pre-trained BERT-based large language monolingual and multilingual models systematically perform better on the English language compared to non-English languages. This disparity exists regardless of the model and task. Figure 1 shows the corresponding results.

This is an alarming finding because this shows that even if the same harmful content is spreading in English and in non-English languages, the disparity will lead to disproportionately less detection of non-English harmful content and thus, more spread on the platforms. This highlights an inequity issue that needs to be addressed.

The code and data for this work are present at https://github.com/claws-lab/multimodality-language-disparity/.

### 2.4. Multimodal models

I seek to build solutions to develop multimodal solutions to develop the next generation of stable and effective models. Multimodality is crucial to study since online content spans multiple content modalities (text, image, videos) and non-content modalities (graph, time, metadata). Different modalities provide complementary signals. This is especially beneficial in the domain of web integrity where bad actors try to camouflage their behavior to avoid detection—it becomes more challenging for bad actors to camouflage signals across multiple modalities.

For example, my work (Kumar et al., 2018b) in e-commerce fake review and reviewer detection has shown that using signals from the user-to-item review graph along with the text of the review and metadata (timestamps) of the review's content leads to significantly better detection performance compared to using either text or graph. The code and data for this work are present at https://cs.stanford.edu/~srijan/rev2/.

**Figure 2:**
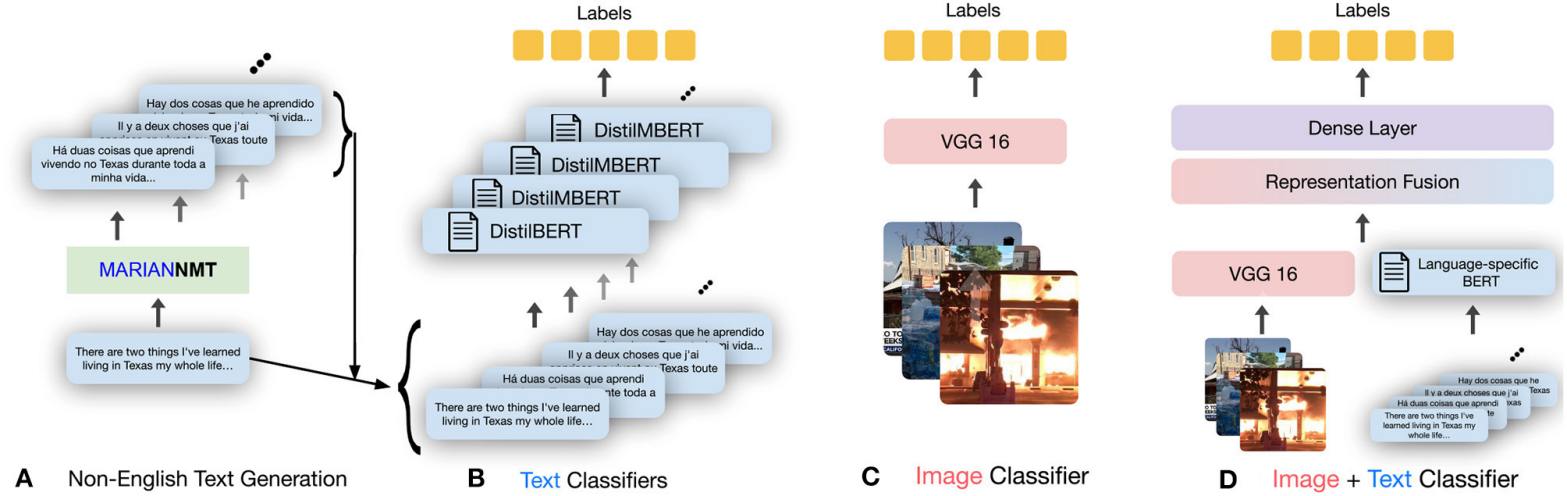
Overview of the adopted multimodal methodology. After using machine translation to obtain high-quality translations of the English text in our datasets **(A)**, the following models are trained: language-specific text-only classification models **(B)** and image-only classification models **(C)**. The multimodal classifier **(D)** fuses the representations obtained from trained text-only and image-only models, and predicts the label based on joint modeling of the input modalities.

Recently, my collaborators and I developed multimodal solutions to bridge the disparity in model performance across languages (Verma et al., 2022b), as described in the previous subsection. The core idea was to use information from other non-textual modalities, such as images, and augment it with the knowledge from the text. Solutions includes multimodal versions of text-based models in multiple languages by creating fusion-based model, as shown in Figure 2. Experimental evaluation showed that including images *via* multimodal learning bridges the performance gap across languages (Figure 1). Specifically, it should be noted that using images improves the model performance in all the languages, but interestingly, the improvement is larger in non-English languages (average 11.5% improvement) compared to the improvement in English (average 5% improvement). The code and data for this work are present at https://github.com/claws-lab/multimodality-language-disparity.

As video-based harmful content is rampant due to the burgeoning prominence of multimedia platforms including YouTube, TikTok, etc., my future research will develop multimodal detection systems that work across platforms, languages, and modalities.

## 3. Robust detection models against adversarial actors by predicting future malicious activities

Bad actors adapt their behavior to fool detectors deployed by platforms to detect them. Current approaches are reactive to adversaries, i.e., models are updated *after* adversaries have changed their behavior. This puts detection models one step behind the adversaries.

Instead, my work is pushing the boundaries by proactively forecasting adversary actions and then improving the robustness and reliability of detection methods against manipulation. The key intuition is the following: if one can predict the different ways in which an adversary can change its behavior, the detection models can be trained to detect these possibilities.

My recent works have innovated one of the first adversarial learning techniques to establish the vulnerabilities of models deployed on the biggest web platforms: Facebook's TIES bad actor detection model (which we showed can fail 26% times by manipulation; the work was funded *via* a Facebook faculty award) (He et al., 2021a), Twitter's Birdwatch misinformation detection platform (Mujumdar and Kumar, 2021), and ban evasion on Wikipedia (Niverthi et al., 2022) (the model is currently being deployed to aid Wikipedia moderators). My future work aims to innovate a suite of benchmarking and robustness-enhancing methods to improve the reliability of detection models.

### 3.1. Adversarial robustness of bad actor detection models

PETGEN is the first-ever framework (He et al., 2021a) to evaluate the trustworthiness, stability, and robustness of bad actor detection models, including the TIES model used at Facebook (Noorshams et al., 2020). To evaluate the bad actor detection models' robustness against adversaries, PETGEN was developed that generates adversarial attacks against the detection model to identify its vulnerabilities. Specifically, a novel adversarial attack text generation framework was created and trained against deep user sequence embedding-based classification models, which use the sequence of user posts to generate user embeddings to detect fraudulent users.

**Figure 3:**
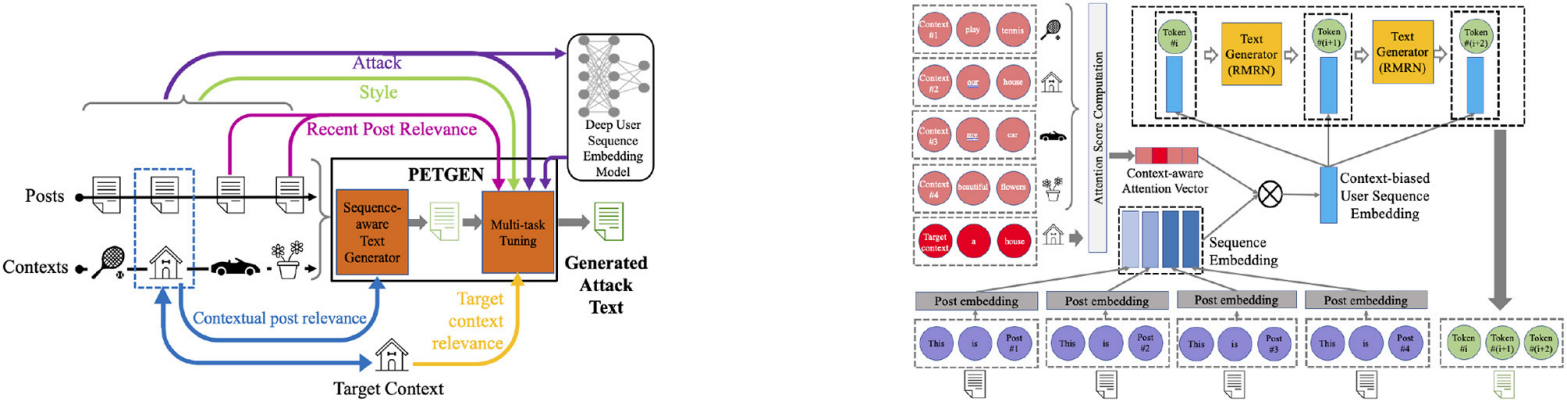
**(Left)** Overview of the proposed architecture, PETGEN: The sequence-aware text generator utilizes the sequence of posts and context to generate text that maintains the contextual post relevance. **(Right)** Then, the multi-stage multi-task learning module fine-tunes the text by different tasks to generate attack text.

The model's architecture overview is shown in Figure 3 left and the sequence-aware text generator component is visualized in Figure 3 right.

This work was the first to show that existing malicious user classification models are incredibly susceptible to adversarial manipulation. The addition of even one adversarial post to the sequence of user posts can flip the prediction label given by the detection model. Notably, the TIES model can be fooled on an average 26% times by simple post-augmentation. This highlights the instability of detection models.

The result shows the existing user sequence classification models are vulnerable to adversarial attacks. Moreover, extensive experiments on Yelp and Wikipedia datasets show that PETGEN can outperform existing strong baselines in terms of the attack performance. Moreover, PETGEN generates text with higher quality, both in terms of quantifiable metrics and as evaluated by human evaluators.

This work paves the path toward the next generation of adversary-aware detection models where we see variants of possible attacks and then incorporate the attack during the fraud detection model development to improve the model's robustness. Adversarial learning enhances the robustness of fraud detection systems for better reliability. The code and data for this work are present at https://github.com/claws-lab/petgen/.

### 3.2. Robustness of bad actor detection models to ban evaders in practice

**Figure 4:**
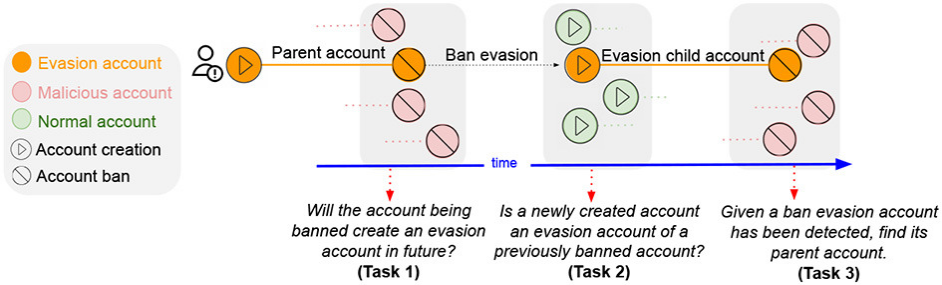
*The ban evasion lifecycle*. Based on the key stages of ban evasion, we formulate three goals: (i) predict future ban evasion, (ii) detect ban evasion soon after the creation of new accounts, and (iii) detection and matching at the time of banning of evasion child account.

Recently, my students and I conducted the first-ever research on ban evasion (Niverthi et al., 2022). Ban evasion is the phenomenon when malicious actors create a new account to continue their harmful activities after getting banned by the platform. Figure 4 shows the lifecycle of a ban evader. This poses a huge challenge to the safety and integrity of the platforms, as even AI-based detection systems are easily evaded.

Since there is no standard dataset, the project curates and presents the first dataset from English Wikipedia to study ban evasion. Wikipedia provides the ground truth of “sockpuppet” groups, i.e., a set of accounts controlled by a single entity. No ban evasion labels are explicitly present, so sockpuppet groups were used to identify at most one ban evasion parent-child pair within the group.

Results showed that malicious actors' evasions can be identified using characteristic linguistic (i.e., what is the content that is added), behavior (i.e., temporal and activity similarity of parent and child account), and activity features (i.e., which pages they edit). Notably, ban evaders fools moderators when the behavioral signatures are changed by the bad actor when using the child account as compared to the parent account.

The dataset has been released for further research. I am now collaborating with the Wikimedia Foundation to develop a ban evasion detection and verification tool to reduce the laborious manual detection process of their moderators. The code and data for this work are present at https://github.com/claws-lab/ban_evasion.

### 3.3. Robustness of multimodal classifiers to content dilutions

Deep multimodal models are being used for several social good applications that involve modeling user-generated data. As such applications become mainstream, it is critical that we study the robustness of multimodal robustness to not just imperceptible perturbations in data but also plausible changes. My recent work (Verma et al., 2022c) takes the first step toward this goal by introducing and generating realistic cross-modal dilutions that successfully highlight the model's vulnerabilities.

The work demonstrated that widely-used fusion-based multimodal classifiers are not robust to cross-modal dilutions—i.e., additions to the original text that describe the corresponding image, and experience a performance drop of over 20 absolute *F*_1_ points across two societal tasks—detecting humanitarian information during crises and detecting emotions. The dilutions are perceived as realistic by human annotators, indicating the low robustness of multimodal classifiers in plausible scenarios where users post additional relevant text than seen in the training corpus. The code and data for this work are present at https://github.com/claws-lab/multimodal-robustness.

## 4. Attributing the impact of harmful content and the role of technology

I seek to create data-driven techniques to establish the role that online harmful content has in causing real harm - such as exacerbating mental health and provoking violence against racial and ethnic minority communities. I then aim to develop intervention solutions to mitigate and prevent the harms. I also seek to understand the sensitivity of recommender systems and how to create stable recommender models that cannot be manipulated by adversaries.

### 4.1. Impact of misinformation on anxiety

**Figure 5:**
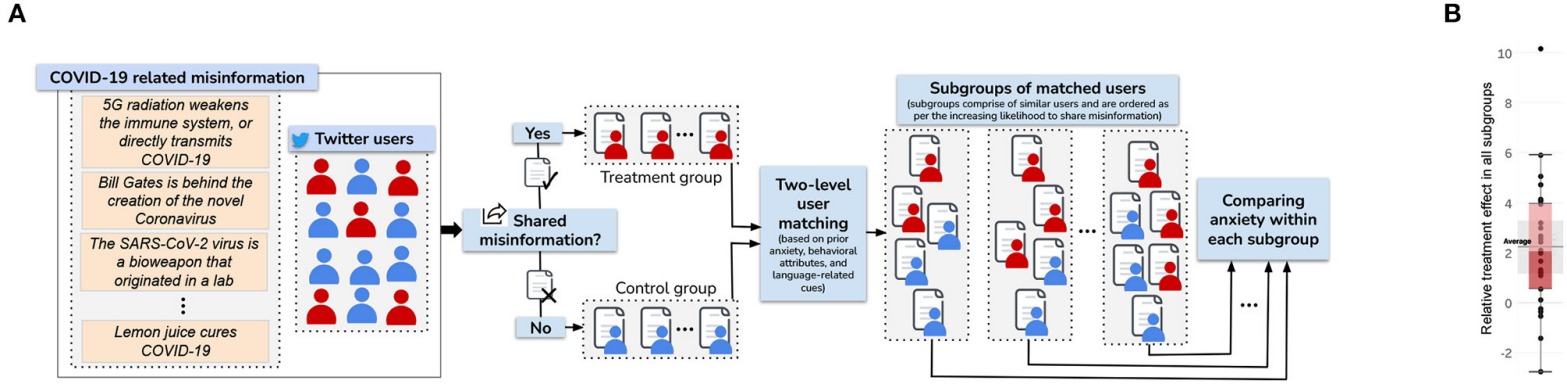
Causal inference methodology **(A)** and the effect of sharing misinformation on experiencing anxiety - overall distribution **(B)**. This figure illustrates our methodology to study the causal effect of sharing misinformation (treatment) on experiencing heightened anxiety (outcome) **(A)**. Users are identified who shared considerable COVID-19 misinformation on Twitter and assign them to the treatment group, while assigning the ones who did not share any misinformation to the control group. Then, a two-level matching strategy is employed to identify similar users across the two groups, using several factors like prior anxiety, other prior mental health indicators, platform-specific behavioral attributes, and language-related cues. Within each subgroup of matched users, the aggregate anxiety levels of treatment and control users are compared using their post-treatment Twitter posts to estimate the effect of sharing misinformation. **(B)** Shows a box and whisker plot of the relative treatment effect across all subgroups. The average, first and third quartiles, and the 95% confidence interval all lie above 0.

Recently, my collaborators and I conducted the first study (Verma et al., 2022a) on the impact of misinformation on the anxiety of users on social media. The work created a novel causal inference framework using social media data, which gives large-scale volume and generalizability over traditional small-scale randomized control trials (RCTs). The pipeline is shown in Figure 5.

The work covered data spanning 18.5 months containing 80 million tweets. Results showed, for the first time, that users who share misinformation (which is a strong signal of consuming it) experience two times additional increase in anxiety compared to similar users who did not share misinformation. Alarmingly, women, racial minorities (Black, Asian, Hispanic), and less educated experience a disproportionately higher increase. This work is the first to shed light on the mental health cost of misinformation, which has long been hypothesized. This work has important practical implications for social media platforms in curbing the adverse psychological impacts of misinformation.

### 4.2. Manipulation of recommender systems

**Figure 6:**
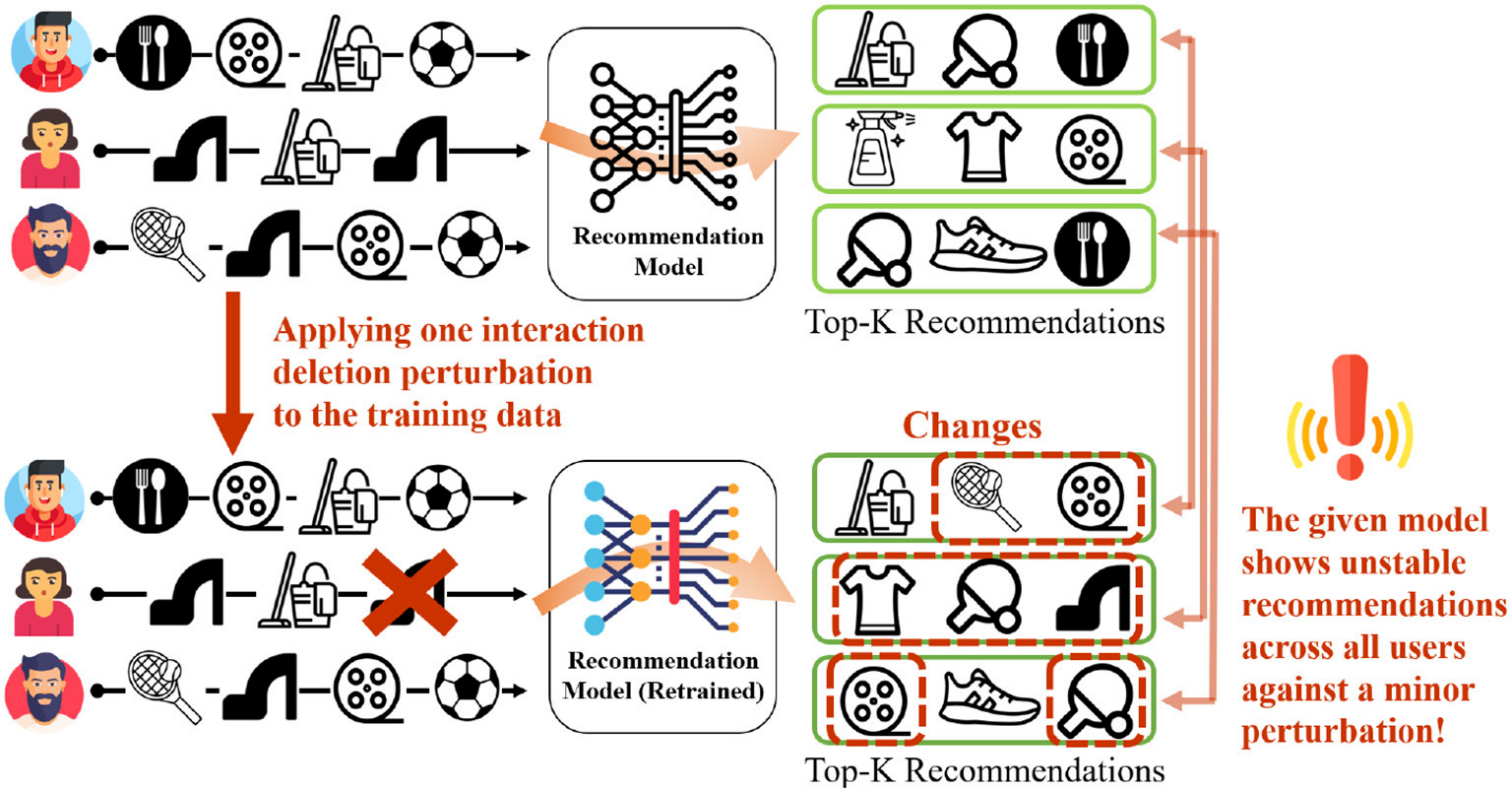
Minor perturbation in the training data of recommender systems can drastically alter the recommendation ranked lists of all the users. Here a perturbation of deleting one user-item interaction is shown.

My recent work (Oh et al., 2022b) illustrates how recommendation systems can be manipulated by adversaries to promote their content online. My collaborators and I created a novel framework to study the impact of insignificant, minor perturbations on the recommendation list of all the users, as shown in Figure 6. Specifically, minor perturbations are injected into the training data and study the impact on the resulting recommendations — the hypothesis is that a model is stable if the outcomes do not change when the perturbations are insignificant.

This work studied the stability of multiple deep recommender systems. Perturbations include insertion, deletion, or replacement of *one* interaction from the training data. Since the training data contain millions of data points, change in a single data point is considered minor and insignificant.

Despite this, results showed that even a single random training data perturbation can drastically alter the recommendations provided to all users. This illustrates the instability of these recommender system models. Notably, the instability was observed to be higher for users for whom recommendations are already inaccurate, thus highlighting a fairness issue. Such instability can be exploited by bad actors to generate unstable and low-quality recommendations to all or subset of users. The code and data for this work are present at https://github.com/claws-lab/casper.

Current and future research aims to build novel solutions to create recommender systems that are stable and investigate when and why undesirable recommendations are made to users.

## 5. Developing mitigation techniques and tools to counter misinformation

I strive to create multi-pronged strategies—empowering professional fact-checkers and journalists, developing interventions, and enabling ordinary users—to mitigate the harmful impact of dangerous online content. This solution requires not only deep technical understanding but also skills to conduct experiments involving human subjects.

### 5.1. Crowdsourced solutions to counter misinformation and hate speech

My recent project (Micallef et al., 2020) collected and studied 8 million COVID-19 tweets, hand-annotated data, and built a language-based detection model using BERT to show that ordinary non-expert users play the most prominent role in countering online misinformation - 96% of all counter misinformation messages are made by ordinary users. However, results showed that 2 out of 3 counter messages are rude and non-evidenced, highlighting the need to empower effective countering strategies at the grassroots level. The code and data for this work are present at http://claws.cc.gatech.edu/covid_counter_misinformation/.

My students and I recently explored the properties of misinformation posts that get countered (Ma et al., 2023). This work focused on answering the following two research questions: (1) “Given a tweet, will it be countered by other users?”, and (2) “If yes, what will be the magnitude of countering it?”. This exploration will help develop mechanisms to guide users' misinformation correction efforts and to measure disparity across users who get corrected. This work created a novel dataset with 690,047 pairs of misinformation tweets and counter-misinformation replies. Experiments showed that several features of tweets that attract social correction, such as anger and impoliteness. The code and data for this work are present at https://github.com/claws-lab/social-correction-twitter.

Most recently, my collaborators and I created a model to generate factual counter-responses to misinformation posts (He et al., 2023) in order to assist ordinary users reply with factual, polite, and countering responses. The work created two novel datasets of misinformation and counter-misinformation response pairs from in-the-wild social media and crowdsourcing from college-educated students. Using annotations on the collected data, poor responses are distinguished from ideal responses that are factual, polite, and refute misinformation. The work proposes MisinfoCorrect, a reinforcement learning-based framework that learns to generate polite, factual, and refuting counter-misinformation responses for an input misinformation post. Quantitative and qualitative evaluation shows that our model outperforms several baselines by generating high-quality counter-responses. The code and data for these work is present at https://github.com/claws-lab/MisinfoCorrect.

Further, my collaborators and I (He et al., 2021b) conducted the longest longitudinal study by collecting and analyzing 206 million tweets, hand-annotated data, and built BERT-based models to establish the prevalence of anti-Asian hate speech on Twitter. Notably, by conducting simulated network experiments, results showed that counterspeech, i.e., speaking up against hate speech, can reduce the probability of neighbors making hate posts. The code and data for this work are present at http://claws.cc.gatech.edu/covid.

### 5.2. Developing tools to counter online misinformation

I seek to develop misinformation mapping tools for experts to track the spread of misinformation and create interventions to prevent its spread further. The goal of these tools is to map the spreaders and consumers of misinformation, provide an easy-to-use interface to professionals to track and visualize the spread in real-time, and then enable them to deliver interventions to the users most vulnerable to the misinformation. These tools will enable faster, accurate, and focused fact-checking by experts, as well as correction of misinformation. These projects are partly funded by the National Science Foundation.

### 5.3. Addressing community violence-provoking health misinformation

Recently, I have received a grant from the U.S. Centers for Disease Control and Prevention (CDC) to study the prevalence, impact, and mitigation of racially- and ethnically-motivated community violence-provoking health misinformation online. Such misinformation tends to harm minority and marginalized communities, who are already victims of discrimination and systematic structural inequities.

Hate speech, bullying, and calls for racially-motivated violence are prevalent on web and social media platforms. Misinformation directed against minority communities may take several forms, ranging from explicit calls for violence against the community to offensive, hateful, and anxiety- and fear-inducing statements against the targeted community. Regardless, these forms of misinformation increase fragmentation and polarization in society. Health misinformation is designed to provoke negative affective emotions (e.g., anger, anxiety), degrading of attitudes (e.g., trust, liking, empathy) toward target community members, and may increase their intention to engage in online and physical harm toward self or others. This adversely impacts the members of the target community both in the online and the physical world. Target community members experience harassment, aggression, trolling, doxing, and racism online, along with physical assaults, abuse, riots, and mob lynchings in the real world. For instance, more than 11,000 incidents of physical violence and online aggression against Asians were reported in the wake of the COVID-19 pandemic. COVID-19 false claims have also led to destruction of property and over 800 deaths. The mental health costs are massive too. Targeted harassment deteriorates the victim's mental health. Thus, it is crucial to understand the prevalence and impact of racially- and ethnically-motivated community violence caused by health misinformation online.

Given the prevalence of Anti-Asian and Anti-Black discrimination and violence in the context of COVID-19 virus spread, we will focus on violence-provoking misinformation targeting these two communities: (1) Anti-Asian and (2) Anti-Black.

This work has four goals: (1) identifying health misinformation that promotes community violence, (2) mapping and measuring the prevalence of community violence-provoking health misinformation across social media platforms, (3) establishing the causal impact of such misinformation on the readers' reactions and intention to engage in harm, and (4) designing mitigation and intervention strategies to reduce the prevalence of such misinformation.

This work is in collaboration with the Anti-Defamation League to develop actionable solutions that will be effective in practice.

## 6. Conclusion and future scope

Together, these four thrusts seek to develop complementary approaches to solve online integrity problems. They create an end-to-end framework to provide holistic solutions from detection all the way to mitigation. The first two thrusts ensure that any detection model that is created is accurate as well as robust. The third thrust aims to estimate the impact that online harms have in the online as well as in the real world. It also helps infer the role of recommender systems in exacerbating harms. And finally, the fourth thrust develops a series of solutions to counter online harms and mitigate its negative impact by empowering professionals as well as ordinary citizens.

Future work directions are plenty in each thrust.

- The research field is still early in terms of creating accurate detections models that work seamlessly across different languages, modalities, and platforms. Current research, including my own, has illustrated promising early research in these overarching directions. Importantly, new benchmark datasets are necessary to create a standardized comparison of the different methods that have been developed. The current lack of standardized benchmarks have made it harder to compare.
- In robustness, new methods are needed that can help estimate how secure and robust detection models are against adversarial manipulation and non-adversarial changes, such as data drift and concept drift. Once robustness is benchmarked, solutions are needed that can improve the robustness of existing models as well as entirely new models that are robust against a variety of manipulation.
- Attributing the impact that AI models, including generative AI models and recommender systems, have on the creation and spread of dangerous content and the reach of bad actors is an open problem. With the new wave of generative AI models (e.g., ChatGPT, Dall-E, etc.), this problem is going to skyrocket as generating harmful content becomes cheaper and it becomes easier to generate believable but fake evidence to support wrong information. Measuring their prevalence and impact is the first step toward developing mitigation solutions.
- Mitigating the impact of online harms is also an open problem. Existing research has tested a small number of mitigation strategies on specific topics. However, a broader understanding of generalizable mitigation strategies does not exist, which remains to be researched. Importantly, when empowering ordinary users to counter misinformation, many questions are unanswered: which countering strategies are more effective; do effective strategies depend on the misinformation spreader's or corrector's demographics; which misinformation should be corrected; when should misinformation be corrected; who should correct the misinformation; if misinformation correction backfires, how to mitigate that?

## Data availability statement

The original contributions presented in the study are included in the article/supplementary material, further inquiries can be directed to the corresponding author.

## Author contributions

The article was written by SK.
